# Alcohol dependence and consumption status are related to smoking status: A cross-sectional study of data from the Japan Society and New Tobacco Internet Survey 2023

**DOI:** 10.18332/tpc/208542

**Published:** 2025-07-31

**Authors:** Kiho Miyoshi, Takahiro Tabuchi, Takashi Miyawaki

**Affiliations:** 1 Graduate School of Home and Economics, Kyoto Women’s University, Kyoto, Japan; 2 Division of Epidemiology, School of Public Health, Graduate School of Medicine, Tohoku University, Sendai, Japan

**Keywords:** alcohol dependence, smoking status, heated tobacco products, dual smokers, AUDIT scores, TDS test

## Abstract

**INTRODUCTION:**

A significant association has been established between tobacco-smoking and alcohol consumption. However, few studies have investigated this association according to tobacco product type. This study aimed to investigate alcohol dependence according to smoking status.

**METHODS:**

The analysis targeted 31465 participants of the Japan Society and New Tobacco Internet Survey 2023, including 19927 never smokers (63.3%), 6545 ex-smokers (20.8%), 2461 cigarette smokers (7.8%), 1496 heated tobacco product (HTP) smokers (4.8%), and 1036 dual smokers (3.3%). Participant characteristics, including sex, age, and body mass index (BMI), were recorded. Alcohol-dependence status was defined as an alcohol use disorders identification test (AUDIT) score of >13. The Tobacco Dependence Screener (TDS) test was conducted to assess nicotine dependence.

**RESULTS:**

Participants had a median age 47 years (interquartile range, IQR: 33–62) and a median BMI of 21.6 kg/m^2^ (IQR: 19.6–24.0). While never smokers showed the lowest AUDIT scores (median: 1), cigarette, HTP, and dual smokers exhibited significantly higher AUDIT scores than never smokers (cigarette: 3, HTP: 4, dual smoker: 4; all p<0.001). Dual smokers showed the highest rate of alcohol dependence (14.9%), followed by HTP smokers (10.7%), cigarette smokers (10.5%), ex-smokers (7.8%), and never smokers (2.2%). In logistic regression analysis, factors related to smoking status – such as number of tobacco products consumed per day, TDS scores, and smoking type – were significantly related to alcohol dependence, along with demographic factors of age and sex. Dual smokers were four times more likely to be alcohol dependent than never smokers (adjusted odds ratio, AOR=4.07; 95% CI: 3.09–5.46).

**CONCLUSIONS:**

Smoking status is significantly associated with alcohol consumption status.

## INTRODUCTION

Nicotine, present in tobacco leaves, and alcohol consumption are both known to cause addiction. Nicotine is an alkaloid that binds to nicotinic acetylcholine receptors^1^, inducing the secretion of dopamine – a neurotransmitter in the brain that produces pleasure – thus causing addiction^2^. Alcohol also activates the dopaminergic nervous system in the brain, resulting in a temporary feeling of pleasure through the brain reward system^3^.

The use of heated tobacco products (HTPs) has been increasing significantly in Japan after 2014. According to the National Health and Nutrition Survey conducted in Japan^4^ in 2022, the current prevalence of tobacco-smoking is 14.8% in the country, with approximately 18 million people reporting smoking habits. The survey also showed that one in three smokers in the country is using HTPs. Global research^5^ has shown that the prevalence of HTP use was highest in Japan and South Korea during 2015–2022, with current and daily HTP use reported to be 4.87%. HTP usage is increasing in Asia and the European and Western Pacific regions, and is projected to continue increasing globally over the coming years^6-8^.

A significant association has been established between tobacco smoking and alcohol consumption. For instance, heavier smoking behaviors are related to higher rates of alcohol dependence^9^, and alcoholic beverages are more likely to trigger cravings for tobacco^10^. Studies regarding the combination of alcohol and cigarette smoking have reported that heavy drinkers with smoking habits are at significantly higher risk of dementia^11^ and throat cancer^12^ than non-smokers or non-drinkers, and non-smoking light drinkers. Therefore, it is important to assess both smoking and alcohol statuses together. However, only few studies in the literature have investigated the association between HTP smoking and drinking habits^13^. Moreover, to our knowledge, no study has investigated the association between both HTP and cigarette smoking (i.e. dual users) and drinking habits.

This study aimed to support smoking and alcohol addiction treatment by investigating the relationship between alcohol dependence and smoking status, including different smoking practices such as HTP-only and dual usage of both HTP and cigarettes.

## METHODS

### Study design, setting, and participants

In this nationwide cross-sectional study, we used alcohol consumption data from the alcohol use disorders identification test (AUDIT)^14^ and smoking status data from the Japan Society and New Tobacco Internet Survey (JASTIS)^15^. The JASTIS is an Internet-based cross-sectional research project that aims to better understand the use of new types of tobacco (HTP) in Japan, as well as investigating the current regulations and health effects. The JASTIS collects data from the pooled panels of an Internet research agency with approximately 2.3 million active panelists (Rakuten Insight Inc., Tokyo, Japan). The data used in this study were from the most recent survey conducted with a sample size of 34000 respondents during 6–27 October 2023. Further details concerning the JASTIS, quality controls, and other policies have been described previously^15^.

### Inclusion and exclusion criteria

This study included data from all respondents of the 2023 JASTIS survey (n=34000), except those whose responses were inconsistent with the information provided in earlier JASTIS surveys. We also excluded straight-lined responses or those that contained discrepancies – for example, if respondents reported themselves to be former users of tobacco products but also indicated that they had never used these products. In addition to these exclusion criteria, we performed an attention check using the item: ‘Please choose the second response from the bottom’, which allowed us to exclude 2535 participants for lack of attention.

### Measurement of exposure: tobacco product use

To evaluate the association between past tobacco product use, combustible cigarette use, HTP use, and combined use compared to never smokers, this study defined specific exposures aimed at clearly differentiating each smoking status. The respondents who were categorized as current tobacco product users were asked: ‘Over the past 30 days, have you used any of the following tobacco products: combustible paper-wrapped cigarettes; self-rolled cigarettes; or heated tobacco products such as IQOS, glo, or Ploom S?’. The response options were ‘Yes’ and ‘No’. Among the current users, the types of products were classified as: combustible cigarettes (paper-wrapped and self-rolled cigarettes) and HTPs (Ploom tech, Ploom tech plus, Ploom S, IQOS, and glo). We differentiated between the respondents who used only combustible cigarettes, those who only used HTPs, and those who used both (dual users). Among those who did not use tobacco products, we separated former users from those who had never used them. As a result, the included respondents were classified as one of the following: never smoker (n=19927), ex-smoker (n=6545), cigarette smoker (n=2461), HTP smoker (n=1496), or dual smoker (n=1036).

### Questionnaires

*Main outcome measures*

We defined self-reported AUDIT^14^ scores as the primary outcome, as the JASTIS survey included the AUDIT. The AUDIT is a self-administered questionnaire that screens for and estimates the consumption of harmful and hazardous alcoholic beverages, according to the World Health Organization (WHO) definition^14^. It comprises 10 questions that yield a score of 0–4. Each respondent’s total score was used to determine their level of alcohol dependence, with scores ≥13 being considered dependent^16,17^.

To determine smoking status, the participants were asked when they started (and stopped) smoking cigarettes and HTPs. We then calculated their smoking histories according to their reported ages. The duration of using HTPs was also estimated. The participants were also asked about the number of cigarette sticks or HTP sticks used per day. Those who answered less than 100 sticks were excluded. The Tobacco Dependence Screener (TDS)^18^ was used to assess nicotine dependence. This survey, which assesses smoking cessation treatment in Japan, was developed to diagnose nicotine dependence from a psychiatric perspective in accordance with the WHO International Classification of Diseases, 10th Edition, and the revised 3rd and 4th editions of the American Psychiatric Association’s Guide to the Classification and Diagnosis of Psychiatric Disorders. It comprises 10 questions, with a score of 1 assigned for ‘yes’ and 0 for ‘no’. Each respondent’s total score for the 10 questions was used to determine their level of dependence. A score ≥5 was considered indicative of nicotine dependence^18^.

### Statistical analysis

All the data were analyzed digitally, using IBM SPSS version 28 (IBM Corp., Armonk, NY, USA). They were analyzed using the Shapiro-Wilk normality test, which failed to identify a normal distribution. Therefore, the data are presented as medians and interquartile ranges (IQRs). The Kruskal-Wallis test and Bonferroni correction were used to assess significant inter-group differences in the participants’ characteristics. Fisher’s exact test and residual analysis were performed to assess the rate of alcohol dependence as indicated by AUDIT scores, based on smoking status. Logistic regression analysis was performed to estimate adjusted odds ratios (AORs) and 95% confidence intervals (CIs) for the prevalence of alcohol dependence, as measured by AUDIT. The dependent variable was alcohol dependence as defined by the AUDIT score, while the independent variables were age, sex, BMI, TDS score, number of sticks used per day, and smoking status (i.e. never smoker, ex-smoker, cigarette smoker, HTP smoker, or dual smokers). Statistical significance was set at p<0.05.

### Ethical considerations

Web-based informed consent was obtained from all the included respondents for the use of their JASTIS data in this study. This study was approved by the Institutional Review Board of Osaka International Cancer Institute (approval number 16110791633) and Tohoku University (approval number 2024-1-517). All the data were anonymized prior to the statistical analyses.

## RESULTS

### Participant characteristics

Table 1 shows the characteristics of participants according to their smoking status. Their median age was 46 years (IQR: 33–62). When grouped by smoking status and type of cigarettes used, never smokers comprised the youngest, followed by HTP smokers, dual smokers, cigarette smokers, and ex-smokers. Never smokers were significantly younger than ex-smokers (p<0.001), cigarette smokers (p<0.001), HTP smokers (p=0.022), and dual smokers (p=0.004). HTP smokers were significantly younger than cigarette smokers (p<0.001) and ex-smokers (p<0.001); however, there was no statistical difference between HTP smokers and dual smokers (p>0.999). Dual smokers were significantly younger than cigarette smokers and ex-smokers (both p<0.001). Ex-smokers were significantly older than cigarette smokers (p<0.001).

**Table 1:** Participant characteristics, Japan, 2023 (N=31465)

*Characteristics*	*All* *n*	*Never smokers*	*Ex-smokers*	*Cigarette smokers*	*HTP smokers*	*Dual smokers*	p*
**Total,** n	31465	19927	6545	2461	1496	1036	
***Gender***	***n***	***n (%)***					
Male	15149	7099 (35.6)	4413 (67.4)	1731 (70.3)	1087 (72.7)	819 (79.1)	<0.001
Female	16316	12828 (64.4)	2132 (32.6)	730 (29.7)	409 (27.3)	217 (20.9)	
***Personal***	***Median (IQR)***						
Age (years)	47 (33–62)	41 (29–59)	58 (45–70)	53 (42–65)	45 (36–55)	46 (35–57)	<0.001
Weight (kg)	58 (50–67)	55 (49–63)	63 (55–71)	62 (55–70)	65 (55–73)	65 (56–73)	<0.001
Height (cm)	163 (157–170)	161 (156–168)	167 (160–172)	168 (161–173)	169 (162–173)	170 (165–174)	<0.001
BMI (kg/m^2^)	21.6 (19.6–24.0)	21.0 (19.2–23.3)	22.8 (20.7–25.0)	22.2 (20.0–24.7)	22.5 (20.3–25.1)	22.3 (20.2–24.7)	<0.001
**Smoking**							
TDS scores				4 (1–7)	4 (1–7)	4 (1–7)	0.308
The number of tobacco sticks per day				12 (8–20)	10 (3–20)	10 (5–15)	<0.001
Smoking history (years)				34 (23–45)	25 (15–36)	26 (15–38)	<0.001
Cigarette smoking history (years)				34 (23–45)	20 (10–30)	10 (5–15)	
HTP smoking history (years)					5 (3–7)	5 (3–7)	
**Nicotine dependence** (%)				45.2	46.7	47.3	0.428

Data were tested using the Shapiro-Wilk normality test, and a normal distribution was not found. Therefore, data are presented as median (IQR) or percentage. The Kruskal-Wallis test, Bonferroni correction, and Fisher’s exact test were used to assess significant intergroup differences in characteristics. HTP: heated tobacco products. BMI: body mass index. TDS: Tobacco Dependence Screener. IQR: interquartile range.

* Statistical significance was set at p<0.05.

Regarding body mass index (BMI), never smokers had the lowest values, followed by cigarette smokers, dual smokers, HTP smokers, and ex-smokers. Never smokers had significantly lower BMI values than cigarette smokers (p<0.001), HTP smokers (p<0.001), dual smokers (p<0.001), and ex-smokers (p<0.001). Although cigarette smokers showed a significantly lower BMI than ex-smokers (p<0.001), there were no significant differences between HTP (p=0.074) and dual smokers (p>0.999). HTP smokers also showed no statistically significant differences in BMI values when compared to ex-smokers (p=0.189) and dual smokers (p=1.000). Dual smokers had significantly lower BMIs than ex-smokers (p=0.024).

There were no significant differences in TDS scores and nicotine dependence rates between cigarette, HTP, and dual smokers (TDS scores, p=0.308; nicotine dependence rate, p=0.428), although almost half of these smokers were diagnosed as nicotine-dependent.

Cigarette smokers used the highest number of sticks per day, followed by HTP and dual smokers (cigarette smokers vs HTP smokers, p<0.001; cigarette smokers vs dual smokers, p<0.001; HTP smokers vs dual smokers, p<0.001).

### Alcohol use status

**Figure 1:**
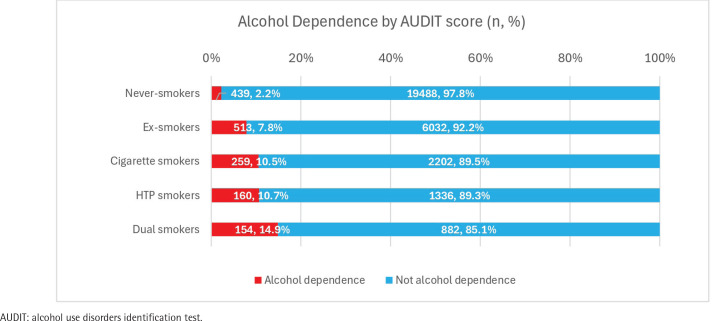
Alcoholism and alcohol dependence rates as measured by AUDIT scores, by smoking status, Japan, 2023 (N=31465)

Figure 1 shows the alcohol dependence rates among the participants, by smoking status as measured via their AUDIT scores. The rate of alcohol dependence showed similar results to those of the AUDIT scores: never smokers comprised the smallest group (2.2% of the total participants), followed by ex-smokers (7.8%), cigarette smokers (10.5%), HTP smokers (10.7%), and dual smokers (14.9%). Approximately 25% of the dual smokers were alcohol dependent.

Table 2 shows the participants’ alcohol consumption status. Never smokers showed the lowest AUDIT scores, followed by ex-smokers, cigarette smokers, HTP smokers, and dual smokers. The AUDIT scores of never smokers were significantly lower than those of cigarette smokers (p<0.001), HTP smokers (p<0.001), dual smokers (p<0.001), and ex-smokers (p<0.001). Ex-smokers showed significantly lower AUDIT scores than HTP (p=0.003) and dual smokers (p<0.001), but no significant difference was found between them and cigarette smokers (p>0.999). Cigarette smokers showed significantly lower AUDIT scores than dual smokers (p<0.001), but no statistically significant difference was found when comparing them to HTP smokers (p=0.110). Even when compared to HTP smokers, dual smokers showed the highest AUDIT scores (p<0.001).

**Table 2:** Alcohol consumption status of the participants, Japan, 2023 (N=31465)

	*All*	*Never smokers*	*Ex-smokers*	*Cigarette smokers*	*HTP smokers*	*Dual smokers*	p*
**Total**, n	31465	19927	6545	2461	1496	1036	
**AUDIT score,** median (IQR)	2 (0–4)	1 (0–3)	3 (1–6)	3 (0–6)	4 (1–7)	4 (2–9)	<0.001
**Alcohol dependence by AUDIT score,** n (%) (% of 1525)	1525 (4.8)	439 (2.2) (28.8)	513 (7.8) (33.6)	259 (10.5) (17.0)	160 (10.7) (10.5)	154 (14.9) (10.1)	<0.001

Data were tested using the Shapiro-Wilk normality test, and a normal distribution was not found. Therefore, data are presented as median (IQR) or percentage. The Kruskal-Wallis test, Bonferroni correction, and Fisher’s exact test were used to assess significant intergroup differences in characteristics. HTP: heated tobacco products. AUDIT: alcohol use disorders identification test.

* Statistical significance was set at p<0.05.

Regarding alcohol dependence by AUDIT score, never smokers showed the lowest rate, at 2.2%. Approximately 10% of the cigarette and HTP smokers, and almost 15% of the dual smokers, were alcohol dependent. After performing a residual analysis, never smokers appeared to be less likely to be alcohol dependent according to AUDIT score (adjusted residual = 28.7). Ex-smokers, as well as cigarette, HTP, and dual smokers, were all more likely to be alcohol dependent (adjusted residual: never smokers = 28.7, ex-smokers = -12.7, cigarette smokers = -13.7, HTP smokers = -10.8, dual smokers = -15.3).

### Contributors to alcohol dependence

The results of our logistic regression analysis are presented in Table 3. The dependent variable was alcohol dependence as defined by the AUDIT score, while the independent variables were age, sex, BMI, TDS score, number of sticks used per day, and smoking status (i.e. never smoker, ex-smoker, cigarette smoker, HTP smoker, or dual smoker).

**Table 3:** Results of logistic regression analysis for alcohol dependence, Japan, 2023 (N=31465)

*Variables*	*AOR*	*95% CI*	p*
Age	0.99	0.98–0.99	<0.001
Sex	0.43	0.38–0.49	<0.001
BMI	1.00	0.99–1.01	0.202
Smoking history	0.99	0.99–1.00	0.101
Number of tobacco sticks use per day	1.01	1.01–1.02	<0.001
TDS score	1.08	1.06–1.11	<0.001
**Smoking status**			
Never smokers (Ref.)	1		
Ex-smokers	2.58	2.18–3.04	<0.001
Cigarette smokers	3.26	2.47–4.30	<0.001
HTP smokers	2.91	2.23–3.81	<0.001
Dual smokers	4.07	3.09–5.36	<0.001

Logistic regression analysis was performed to estimate adjusted odds ratios (AORs) and confidence intervals (CIs) for the prevalence of alcohol dependence, as measured by the AUDIT. These models were adjusted for potential cofounders including age, gender, BMI, smoking history, the number of tobacco sticks used per day, TDS score, and smoking status. BMI: body mass index, HTP: heated tobacco products. TDS: Tobacco Dependence Screener. AUDIT: alcohol use disorders identification test.

* Statistical significance was set at p<0.05.

In logistic regression analysis, factors related to smoking status – such as the number of tobacco products consumed per day, TDS scores, and smoking type – were found to be significantly related to alcohol dependence, in addition to the demographic factors of age and sex (the number of tobacco products consumed per day: AOR=1.01; 95% CI: 1.01–1.02; TDS scores: AOR=1.08; 95% CI: 1.06–1.10; cigarette smokers: AOR=3.26; 95% CI: 2.47–4.30; HTP smokers: AOR=2.91; 95% CI: 2.23–3.81; dual smokers: AOR= 4.07; 95% CI: 3.09–5.46). Smoking type was the strongest contributor to alcohol dependence, with dual smokers being four times more likely to be alcohol dependent than never smokers.

## DISCUSSION

This novel study investigates the association between alcohol consumption status, as defined by the AUDIT survey, and smoking status while accounting for new modalities, such as HTP and dual smoking. We found that AUDIT scores were significantly higher in our cohort of HTP, dual, and cigarette smokers when compared to those of never smokers. The rate of alcohol dependence was low in never smokers, while approximately 10% of the cigarette and HTP smokers, and approximately 15% of the dual smokers, were alcohol dependent according to the AUDIT criteria. After performing an adjusted logistic regression analysis, dual smoking was found to represent a significant contributor to alcohol dependence, whereas never smokers were significantly less likely to be alcohol dependent. HTP and cigarette smokers were both almost three times more likely to be alcohol dependent versus never smokers, while dual smokers were four times more likely.

In this study, both AUDIT scores and rates of alcohol dependence were significantly higher among participants who had smoked tobacco products at some point versus those who had never smoked. Several studies have reported an association between cigarette and/or HTP smoking and alcohol consumption. A previous study reported that cigarette and HTP smokers exhibit a significantly higher rate of drinking, as well as higher intakes of alcohol – and that alcohol intake correlates with daily nicotine consumption among HTP smokers^13^. These results are similar to those of the present study; however, this is the first study, to our knowledge, to investigate the association between alcohol consumption and both HTP and cigarette use.

Several studies have investigated the association between alcohol consumption and smoking. Prior research has shown that alcohol increases cravings for tobacco smoking in both men and women^19^. It has also been reported that alcoholic beverages enhance the taste of cigarette smoke^20^. Additionally, alcoholic beverages are associated with a higher likelihood of smoking cravings^10^. Therefore, alcohol consumption may trigger smoking. One study that analyzed the interaction between nicotine and alcohol claimed that pre-exposure to nicotine increased self-administration of alcohol and decreased alcohol-induced dopamine responses. The authors reported that this blunted dopamine response was caused by increased inhibitory synaptic transmission onto dopamine neurons^21^. Therefore, nicotine exposure may increase alcohol consumption.

In this study, we found that the dual use of HTP and cigarettes was significantly associated with alcohol dependence compared to the use of either product alone or having never smoked tobacco in any form. This may be because HTP contains propylene glycol, acetol, and glycerol^22,23^, all of which are rarely found in cigarettes. These substances may influence the interaction between nicotine and alcohol in a manner that makes it more likely for dual smokers to develop alcohol dependence. However, little evidence is currently available regarding the mechanisms of dual tobacco use and alcohol dependence. Therefore, future research should investigate the mechanisms underlying dual tobacco product use and alcohol dependence.

Several health-related impacts of the combination of alcohol consumption and tobacco-smoking have previously been reported. Studies on the combination of alcohol consumption and cigarette smoking have found that heavy drinkers with smoking habits have a significantly higher risk of developing dementia^11^ and throat cancer^12^. Moreover, one systematic review and meta-analysis focused on oral squamous cell carcinoma (OSCC)^24^, showed that the concurrent consumption of alcohol and tobacco (both smoked and smokeless) significantly increased the odds of developing OSCC. For esophageal squamous cell carcinoma (SCC)^25^, the consumption of fruits and vegetables is associated with a reduced risk of developing the malignancy^25^; however, these beneficial effects cannot fully offset the harmful effects of tobacco and alcohol use. Most smokers consume fewer fruits and vegetables in general than non-smokers^10,13,26,27^, indicating that the risk of developing SCC may be compounded even further in this demographic population. The cessation of smoking and drinking should therefore be prioritized to most effectively reduce the burden of SCC. Furthermore, compared with the use of either product alone or total abstinence from tobacco use, the dual use of electronic nicotine delivery systems (ENDS) and combustible tobacco is significantly associated with incident respiratory symptoms. The combined use of ENDS and tobacco smoking may therefore result in additive or synergistic pathologies^28^.

This study revealed the combined risks of HTPs/cigarette smoking and alcohol drinking. Regarding tobacco-smoking and alcohol cessation, one meta-analysis has shown that smoking cessation interventions during addiction treatments were associated with a 25% increased likelihood of long-term abstinence from alcohol and illicit drug use^29^. A Japanese trial reported that patients with alcohol dependence who quit smoking had a higher success rate in achieving sobriety^30^. Therefore, it may be important to determine not only smoking status, but also the type of tobacco used when treating alcohol dependence. Understanding the smoking status and types of tobacco products used is also of general clinical relevance for such patients. This study suggests that public health campaigns offering a combination of smoking (cigarette, HTPs, or both) and alcohol cessation programs is important, particularly for dual smokers.

### Limitations

This study has some limitations. First, we did not consider the number of cigarettes and HTPs consumed among dual smokers. Potential confounders such as socioeconomic status and family history of addiction were also not considered. Additionally, HTPs are of various types, including IQOS and glo, but no comparison of the different types of HTPs was conducted. Second, the study population was Japanese; therefore, our results may not be generalizable to other populations. Moreover, since the determination of alcohol dependence by AUDIT differs not only in Japan but also around the world, there may be regional differences in the interpretation of the results. Third, all participants were recruited from all over Japan, but they were a willing group to be surveyed and thus may be subject to bias. Fourth, this survey was conducted using self-reported questionnaires, which may introduce biases such as reporting, memory, and cognitive biases. Fifth, because this was a cross-sectional study, causal relationships could not be determined. Future longitudinal or mechanistic studies are warranted to address causality and underlying pathways. Despite these limitations, we could identify alcohol dependence according to smoking status, in HTP as well as dual smokers.

## CONCLUSIONS

Our results indicated that consumers of HTPs, as well as combustible cigarettes and dual users, had a higher prevalence of alcohol dependence than never smokers, after adjusting for a range of confounders. Nicotine and alcohol dependence may therefore be associated. Moreover, our results suggest that HTPs use may promote alcohol usage, in a similar manner to cigarette smoking. The combination of cigarette smoking and alcohol consumption increases the risk of negative health effects such as throat cancer, and concerns have been raised regarding the negative health effects of combined alcohol consumption and HTP use, both alone and in combination with traditional cigarettes. Further research is warranted to confirm our results and further investigate the longitudinal associations between HTP use, dual use of both HTPs and cigarettes, and alcohol dependence.

## Data Availability

The data supporting this research are available upon reasonable request from T. Tabuchi.
